# Comparative evaluation of the diagnostic and prognostic performance of CNSide™ versus standard cytology for leptomeningeal disease

**DOI:** 10.1093/noajnl/vdae071

**Published:** 2024-05-10

**Authors:** Haley R Appel, Muni Rubens, Mukesh Roy, Rupesh Kotecha, Matthew D Hall, Minesh P Mehta, Alexander Mohler, Zhijian Chen, Manmeet S Ahluwalia, Yazmin Odia

**Affiliations:** Department of Radiation Oncology, Miami Cancer Institute, Baptist Health South Florida, Miami, Florida, USA; Department of Clinical Informatics, Miami Cancer Institute, Baptist Health South Florida, Miami, Florida, USA; Department of Clinical Informatics, Miami Cancer Institute, Baptist Health South Florida, Miami, Florida, USA; Department of Radiation Oncology, Miami Cancer Institute, Baptist Health South Florida, Miami, Florida, USA; Department of Translational Medicine, Herbert Wertheim College of Medicine, Florida International University, Miami, Florida, USA; Department of Radiation Oncology, Herbert Wertheim College of Medicine, Florida International University, Miami, Florida, USA; Department of Radiation Oncology, Miami Cancer Institute, Baptist Health South Florida, Miami, Florida, USA; Department of Radiation Oncology, Herbert Wertheim College of Medicine, Florida International University, Miami, Florida, USA; Department of Radiation Oncology, Miami Cancer Institute, Baptist Health South Florida, Miami, Florida, USA; Department of Radiation Oncology, Herbert Wertheim College of Medicine, Florida International University, Miami, Florida, USA; Division of Neuro-Oncology, Department of Medical Oncology, Miami Cancer Institute, Baptist Health South Florida, Miami, Florida, USA; Division of Neuro-Oncology, Department of Medical Oncology, Miami Cancer Institute, Baptist Health South Florida, Miami, Florida, USA; Division of Neuro-Oncology, Department of Medical Oncology, Miami Cancer Institute, Baptist Health South Florida, Miami, Florida, USA; Department of Translational Medicine, Herbert Wertheim College of Medicine, Florida International University, Miami, Florida, USA; Division of Neuro-Oncology, Department of Medical Oncology, Miami Cancer Institute, Baptist Health South Florida, Miami, Florida, USA

**Keywords:** cerebral spinal fluid, CNSide^TM^, cytology, Leptomeningeal disease

## Abstract

**Background:**

This retrospective study compares the real-world performance of cerebrospinal fluid (CSF) CNSide™ versus cytology in leptomeningeal disease (LMD).

**Methods:**

Consecutive patients with suspected LMD who underwent lumbar punctures for CSF cytology and CNSide™ from January 2020 to December 2022 were reviewed. LMD was classified by EANO criteria. Descriptive statistics, confusion matrix, Kaplan–Meier curves, and Cox proportional regression were used.

**Results:**

Median age for 87 evaluable patients was 63 years (range: 23–93); 82 (94%) met EANO criteria for possible/probable/confirmed LMD (EANO/LMD). The commonest primary cancers were breast (36,44.0%) and lung (34,41.5%). Primary lung harbored actionable mutations in 18 (53.0%); primary breast expressed hormone receptors in 27 (75%), and HER2 amplification in 8 (22%). Uncontrolled systemic disease was detected in 35 (40%), while 25 (46%) received systemic therapy with medium/high CNS penetrance at LMD diagnosis. The median time from initial cancer to LMD diagnosis was 31 months (range: 13–73). LMD was confirmed by CSF cytology in 23/82 (28%), all identified by CNSide™. CNSide™ identified 13 additional cases (36/82, 43.9%), increasing diagnostic yield by 56.5%. Median overall survival (mOS) was 31 weeks (95%CI: 21–43), significantly worse for CNSide™ positive versus negative: 4.0 versus 16.0 weeks, respectively (HR = 0.50, *P* = .010). While survival since LMD diagnosis did not differ by histology, time to LMD diagnosis from initial cancer diagnosis was longer for breast (48.5 months, IQR: 30.0–87.5) versus lung (8 months, IQR:0.5–16.0) cohorts. mOS was longer for patients eligible for intrathecal chemotherapy (HR: 0.189, 95%CI: 0.053–0.672, *P* = .010).

**Conclusions:**

This retrospective, real-world analysis of CNSide™ showed increased sensitivity versus cytology and provided clinically relevant molecular CSF analyses.

## Lay Summary

When cancer spreads to the fluid surrounding the brain and spinal cord, it is called leptomeningeal disease. The usual method to check for this is by directly examining the fluid for cancer cells, a process known as cytology. In this study, the authors wanted to see how well a new test called CNSide™ compares to the standard cytology in diagnosing this spread of cancer around the brain and spinal cord. To do this, they looked at results from 87 patients who had both tests completed over a 2-year period. Most of these patients had breast or lung cancer, and some had cancer that was not under control elsewhere in their bodies. Of these patients, 23 were diagnosed with the spread of cancer around the brain and spinal cord by standard cytology. CNSide™ was able to diagnose an additional 13 patients, boosting the overall detection rate by more than half.

**Key Points**

The retrospective study shows the real-world use of CNSide™ in leptomeningeal disease.
CNSide™ yielded higher sensitivity versus cytology
CNSide™ provided clinically relevant molecular CSF analyses.

**Importance of the Study**

Leptomeningeal disease (LMD) is a rare, but highly morbid and fatal complication affecting 5-10% of cancer patients. CSF cytology still remains the diagnostic gold standard with the highest specificity, however, malignant cells are detected in only 50–67% of initial lumbar punctures. LMD diagnosis is made radiographically, clinically, or by cytology of cerebrospinal fluid (CSF) based on EANO criteria, defined as definite, probable, possible, or no evidence of LMD. In a single-institution series, CNSide™ increased the diagnostic yield by 56.5%. CNSide™ also showed increased sensitivity relative to standard cytology and increased specificity relative to EANO criteria and provided clinically relevant, -cell-based molecular and -cell-free DNA analyses.

Leptomeningeal metastatic disease (LMD) is a rare, but highly morbid and fatal complication affecting 5–15% of cancer patients and with rising incidence.^1–3^ LMD occurs when cancer spreads to the leptomeninges or subarachnoid space by hematogenous spread, direct infiltration from solid brain metastases, perineural, or perivascular invasion, or seeding of cancer cells after neurosurgical interventions.^1,4,5^ Once seeded in the meninges, cancer cells can circulate along the meningeal and ependymal surfaces or via cerebrospinal fluid (CSF) flow, with a preference for settling in regions of slow CSF flow, and gravity-dependent locations, such as the posterior fossa, basilar cisterns, and lumbar cistern.^2–4^

LMD diagnosis is confirmed by the detection of malignant cells in the CSF or suggested by clinical and/or neuroimaging findings in patients with metastatic disease.^2^ The diagnosis is often quite challenging due to limited sensitivity with initial cytology (50–67%) and MRI (75%), as well as high variability of clinical presentation.^2,3^ Clinical symptomatology is nonspecific and includes headache (from meningeal irritation), cranial nerve palsies, altered mental status, bowel or bladder dysfunction, extremity numbness and weakness, and symptoms of elevated intracranial pressure from LMD-induced irregularities in CSF dynamics.^2,3^ Using joint European Association of Neuro-Oncology and European Society of Medical Oncology [EANO-ESMO] criteria, LMD diagnosis is defined as type I (confirmed malignant cells in cytology), and type II (lack of verification from cytology).^2^ Neuroimaging findings further classify LMD into different categories, linear disease (type A), nodular disease (type B), both linear and nodular (type C), and no imaging findings or hydrocephalus (type D).^2^ This classification broadens LMD definition as confirmed, probable, possible, or lack of evidence (Table 1), providing guidance on treatment choice with reasonable confidence for “confirmed and probable” cases versus additional work-up advised for “possible or lack of evidence” cases.^2^ CSF cytology remains the diagnostic gold standard for LMD with the highest specificity, however with low sensitivity, as malignant cells are detected in only 50–67% of initial lumbar punctures, increasing to 80–90% only after 3 serial lumbar punctures.^2,3^ Although minimally invasive, lumbar puncture is associated with potential complications including postprocedural headache, cranial neuropathies, nerve root irritation, low back pain, stylet-associated problems, infections, bleeding, and cerebral and spinal herniation.^6^ In addition to these potential effects, procedural time and patient reluctance further limit the feasibility of completing multiple spinal taps in the outpatient setting.^7^ Furthermore, standardization of collection protocols are needed as inappropriate sampling such as limited quantity of CSF retrieved, and inaccurate technique can lead to incomplete analysis reducing the amount of accurate information obtained.^7^

**Table 1.** EANO Diagnostic Criteria for Leptomeningeal Metastasis Disease (LMD)

		Cytology/ biopsy	MRI	Confirmed	Probable^a^	Possible^a^	Lack of evidence^b^
Type I: positive CSF cytology or biopsy	IA	+	Linear	+	NA	NA	NA
	IB	+	Nodular	+	NA	NA	NA
	IC	+	Linear + nodular	+	NA	NA	NA
	ID	+	Hydrocephalus	+	NA	NA	NA
	ID	+	Normal	+	NA	NA	NA
Type II: clinical findings and neuroimaging only	IIA	− or equivocal	Linear	NA	With typical clinical signs	Without typical clinical signs	NA
	IIB	− or equivocal	Nodular	NA	With typical clinical signs	Without typical clinical signs	NA
	IIC	− or equivocal	Linear + nodular	NA	With typical clinical signs	Without typical clinical signs	NA
	IID	− or equivocal	Hydrocephalus	NA	NA	With typical clinical signs	Without typical clinical signs
	IID	− or equivocal	Normal	NA	NA	With typical clinical signs	Without typical clinical signs

Used with permission and modified from Le Rhun et al (2017)^1^.

EANO: European Association of Neuro-Oncology.

Type A: LMD with typical linear MRI abnormalities; type B: LMD with nodular disease; type C: LMD with both linear and nodular disease; type D: LMD without MRI abnormalities (except hydrocephalus).

CSF, cerebrospinal fluid; LMD, leptomeningeal metastasis disease; MRI, magnetic resonance imaging; NA, not applicable.

^a^ Requires a history of cancer with a reasonable risk of LM and consideration of alternative diagnoses.

^b^ Including in patients with a history of cancer.

Currently, the available diagnostic modalities for LMD do not provide reliable information on disease burden or (early) response to treatment.^3^ Recent molecular technological advances provide informative analysis of biofluids with the liquid biopsy, an alternative to tissue biopsy that involves the sampling of body fluids, such as CSF, for molecular components released from cells.^8,9^ Cell-free DNA (cfDNA) are short-fragmented DNA molecules in the plasma, and when released from tumor cells by apoptosis or necrosis, are termed circulating tumor DNA (ctDNA).^3,10^ These molecular tools provide the opportunity to identify specific and potentially actionable therapeutic targets and aid in early diagnosis, assessing treatment response, and monitoring for disease recurrence.^3,8,11^ Tumor-specific genomic abnormalities can be detected in ctDNA using next-generation sequencing (NGS) techniques or polymerase chain reaction (PCR)-based options, and have demonstrated a strong concordance with the genomic profile of malignant tissue, making ctDNA a potentially powerful biomarker.^3,11^

Given the poor sensitivity of standard cytology in diagnosing LMD, the primary aim of this study was to compare the real-world diagnostic and prognostic yield of CNSide^TM^ assay (Biocept Inc.) versus standard cytology. The CNSide^TM^ assay is a novel combination of cell-based and cell-free assays to provide quantitative analysis utilizing proprietary technology.^12,13^ Given the innovative approaches in genomic testing, such as using cfDNA in the CSF, this retrospective analysis also hoped to gain further understanding of prognostic features in patients with LMD by analyzing tumor cell (TC) count, cell density (TC/mL), and molecular features of circulating tumor cells (CTCs) in the CSF assays. The potential to diagnose and characterize CNS disease, replace, or complement ambiguous imaging studies, and monitor treatment response in a minimally invasive manner provide exciting opportunities for LMD detection and treatment.

## Methods

### Population

Consecutive patients at Miami Cancer Institute or Baptist Health Hospital, part of Baptist Health South Florida with suspected LMD from January 2020 to December 2022 who underwent lumbar punctures for CSF cytology and CNSide™ analyses were evaluated. LMD diagnosis was defined by EANO-ESMO criteria.^2^ As the study was retrospective in nature, the requirement for informed consent was waived by the institutional IRB review.

### CSF Assays

CSF cytology was analyzed as per standard practice. CSF tumor cells were also captured and characterized using CNSide^TM^ assay. CSF collected at the time of lumbar puncture was placed into CNSide^TM^ collection tube stabilized at ambient temperature. In the CLIA laboratory CSF-TCs are isolated using a proprietary mouse anti-human antibody cocktail with secondary biotinylated anti-mouse Ig.^13^ All CSF cells are then captured in a streptavidin-coated micro-fluidic channel and fluorescently labeled with DAPI, streptavidin, keratin, and CD45 to discriminate nucleated CSF-TCs that are SA + and cytokeratin+, but CD45 negative.^13^ CSF-TCs are digitally imaged, localized and counted with specific X–Y coordinates in the channel that allow further characterization with biomarker analysis, including fluorescence in situ hybridization (FISH) for rearrangements, copy number variations, and immunocytochemistry (IHC) for proteins and enumeration.^12,13^ For cfDNA, once in the collecting tube, DNA is isolated for NGS.^12^

### Statistical Analysis

All statistical analyses were done using SAS 9.4 (SAS Inc.). Initially, we compared EANO criteria (confirmed, probable, possible, and lack of evidence) by both cytology and CNSide™ to identify their distribution. Test characteristics, such as sensitivity, specificity, positive predictive value, and negative predictive value, were estimated for CNSide™ (cytology as gold standard) and cytology (CNSide™ as gold standard). Then we assessed the concordance between receptor identification by Biocept and primary cancer samples, then stratified by lung cancer and breast cancer using either Chi-squared test or Fisher’s exact test. We compared Biocept CSF volume between CNSide™ positive and negative groups using Mann–Whitney *U* test. We created Kaplan–Meier curves to compare overall survival between EANO-positive (confirmed, probable, possible) versus negative (lack of evidence), CNSide™ positive versus negative, as well as cytology positive and negative. We also compared overall survival between three groups: CNSide™ positive and cytology negative versus CNSide™ and cytology negative versus CNSide™ and cytology positive. We also compared overall survival between CNSide™ positive breast cancer and lung cancer patients. Finally, Cox proportional regression was performed to assess the association between cell density by CNSide™ and survival. All tests were two-sided and statistical significance was set at *P* < .05.

## Results

A total of 87 patients with a median age of 63 years (range, 23–93 years) were evaluated, of which 82 (94%) patients met EANO criteria for LMD: confirmed (*n* = 23, 26.4%), possible (*n* = 40, 46.0%), and probable (*n* = 19, 21.8%). Among the EANO-positive cases, 33 (40.2%) had uncontrolled systemic disease and 25 (30.5%) patients were on systemic therapy with medium/high CNS penetrance.^14^ The median time (IQR) from initial cancer diagnosis to LMD was 31 (21–43) months, statistically longer for primary breast cancer cohorts compared to lung cancer cohorts: 48.5 (30.0–87.5) and 8 (0.5–16.0) months, respectively (*P* < .001). The cohort was predominantly female given the prevalence of primary breast diagnoses. Table 2 shows the demographics at LMD diagnosis.

**Table 2.** Demographics at LMD Diagnosis

Age	
Minimum	23
Maximum	93
Median	63
Gender, n (%)	
Male	21 (24%)
Female	66 (76%)
	Female
Race (ethnicity), *n* (%)	
White (Non-Hispanic)	22 (25%)
White (Hispanic)	56 (64%)
Black	4 (5%)
Other	5 (6%)
Cancer type, *n* (%)	
Breast	39 (45%)
Lung	36 (41%)
Esophagus	3 (3%)
Ovarian	2 (2%)
Astrocytoma	1 (1%)
Endometrial	1 (1%)
Gastric	1 (1%)
Melanoma	1 (1%)
Penile	1 (1%)
Prostate	1 (1%)
Unknown primary	1 (1%)
Tumor status, *n* (%)	
Controlled	39 (45%)
Uncontrolled	35 (40%)
De novo	11 (13%)
Unknown	2 (2%)
Prior craniotomy, *n* (%)	
Yes	13 (15%)
No	74 (85%)
Months from initial cancer	
Minimum	0
Maximum	349
Median	33
Tumor status, *n* (%)	
Controlled	39 (45%)
Uncontrolled	35 (40%)
De novo	11 (13%)
Unknown	2 (2%)

**Figure 1.**
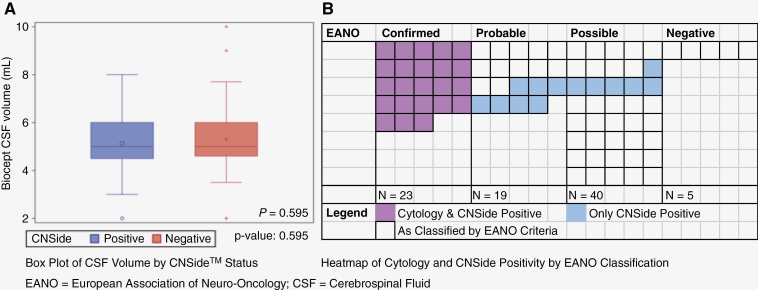
CNSideTM sensitivity by CSF volume and EANO Criteria. (A) A box plot of CSF volume (ml) was not statistically different between CNSide positive or negative status. (B) Color map of cytology and CNSide positive or CNSide positive only among the suspected cases of leptomeningeal metatases categorized by EANO criteria.

LMD was diagnosed by CSF cytology (EANO confirmed) in 23/82 (28%) cases, all confirmed by CNSide™. An additional 13 cases were identified by CNSide™, increasing diagnostic yield by 56.5% for a total of 46/82 (43.9%). Among the 19 EANO probable cases, CNSide™ was positive for 7 (36.8%). Among the 40 EANO possible cases, CNSide™ was positive for 6 (16.0%) (Table 3). Figure 1B shows a heatmap of CNSide-positive cases across EANO definite, probable, possible, and negative status. Test characteristics showed that standard CSF cytology had an expected 100% specificity, but sensitivity was only 70% compared to CNSide™ (Table 3). CSF volume collected ranged from 2 to 10 cc. Figure 1A shows the median (range) CSF volume by CNSide^TM^ status, suggesting no statistical difference between positive (5.0 (2.0–8.0) ml) or negative (5.0 (2.0–10.0) ml) samples by CNSide cell count analysis (*P* = 0.595).

**Table 3.** Comparison of CNSide™ with Cytology Based on EANO Criteria

EANO classification	Positive			Negative
	Confirmed	Probable	Possible	Lack of evidence
Cytology positive Cytology negative	23 (100%) 0 (0%)	0 (0%) 19 (100%)	0 (0%) 40 (100%)	0 (0%) 5 (100%)
CNSide™ positive CNSide™ negative	23 (100%) 0 (0%)	12 (63%) 7* (37%)	34 (84%) 6* (16%)	0 (0%) 5 (100%)

EANO criteria	Cytology positive (*N* = 23)	MRI + C/F (*N* = 19)	MRI (*N* = 40)	No findings (*N* = 5)
Test Characteristic	Statistic estimate		95% Confidence interval	
Sensitivity	63.9%		48.2–79.6%	
Specificity	100%		100%	
PPV	100%		100%	
NPV	80.0%		69.8-89.5%	

EANO: European Association of Neuro-Oncology.

^*^Thirteen additional cases detected by CNSide™ among EANO “probable” and “possible” cohorts.

Commonest primary histologies in the EANO-positive cohort were breast (*n* = 36, 43.9%) and lung (*n* = 34, 41.5%). Among primary breast cancers, 30 (83.3%) expressed ER, 22 (61.1%) expressed PR, and in 8 (22.2%) had HER2 alterations. Among primary lung cancers, 17 (50.0%) cases harbored actionable molecular alterations (we should specify which ones, here). Table 4 shows the concordance between receptor status by CNSide™ versus primary tissue analyses for the whole cohort as well as lung versus breast cancer. Of note, CNSide™ detected a gain in KRAS (2, 5.6%) and EGFR (3, 8.3%) compared to the primary lung cancer biopsy, while a gain in ER (9, 23.1%), PR (3, 7.7%), and HER2 (2, 5.1%) and a loss of HER2 (3, 7.7.%) were noted compared to the primary breast cancer biopsy. Subgroup analyses were limited by sample size, but molecular profiles for all histologies and survival curves for the breast cases among available CNS metastasis surgical samples and the corresponding CSF CNSide^TM^ molecular subgroups in our cohort are listed in Supplementary Tables 1 and 2.

**Table 4.** Concordance Between Receptor Identification by Biocept and Tissue for Overall Sample, Lung Cancer, and Breast Cancer

Whole sample		
BFAR/KRAS	Concordance	6 (6.9%)
	Discordance	3 (3.5%)
	Loss	0 (0%)
	Gain	3 (3.5%)
EGFR	Concordance	5 (5.8%)
	Discordance	3 (3.5%)
	Loss	0 (0%)
	Gain	3 (3.5%)
ER	Concordance	6 (6.9%)
	Discordance	9 (10.3%)
	Loss	0 (0%)
	Gain	9 (10.3%)
PR	Concordance	5 (5.8%)
	Discordance	3 (3.5%)
	Loss	0 (0%)
	Gain	3 (3.5%)
HER2	Concordance	12 (13.8%)
	Discordance	5 (5.7%)
	Loss	3 (3.5%)
	Gain	2(2.3%)
Lung		
BFAR/KRAS	Concordance	6 (16.7%)
	Discordance	2 (5.6%)
	Loss	0 (0%)
	Gain	2 (5.6%)
EGFR	Concordance	5 (13.9%)
	Discordance	3 (8.3%)
	Loss	0 (0%)
	Gain	3 (8.3%)
Breast		
ER	Concordance	6 (15.4%)
	Discordance	9 (23.1%)
	Loss	0 (0%)
	Gain	9 (23.1%)
PR	Concordance	5 (12.8%)
	Discordance	3 (7.7%)
	Loss	0 (0%)
	Gain	3 (7.7%)
HER2	Concordance	11 (28.2%)
	Discordance	5 (12.8%)
	Loss	3 (7.7%)
	Gain	2 (5.1%)

**Figure 2.**
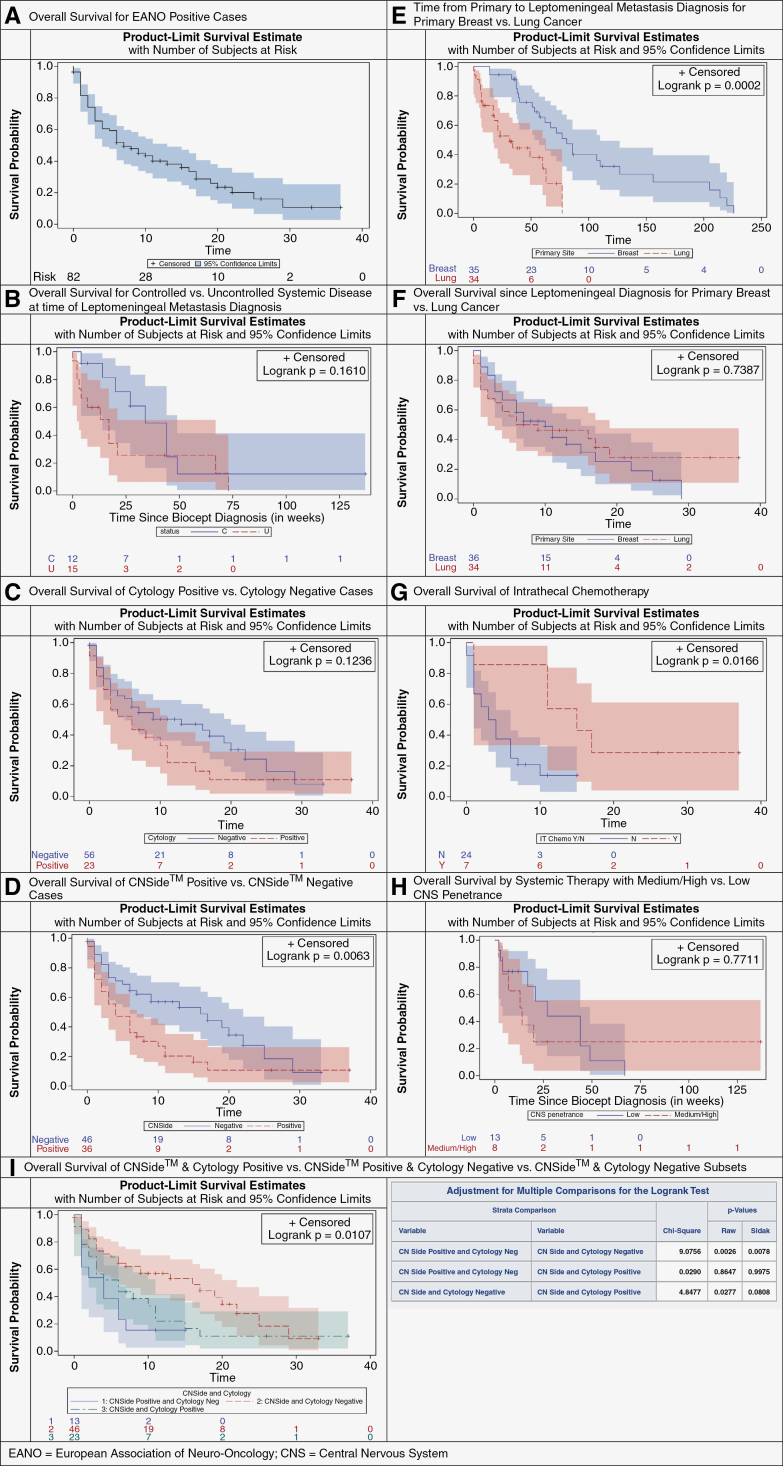
Overall survival by Kaplan–Meier. Kaplan–Meier overall survival curves for various subgroups and comparisons in our leptomeningeal metastases (LMD) cohort, including overall survival for the EANO-positive (confirmed, probable, possible) cohort (A), by status of systemic disease (B), cytology positive and negative (C), CNSide™ positive versus negative (3D), candidacy for intrathecal chemotherapy (G), and CNS penetrance of systemic therapy at the time of LMD diagnosis (H). We also compared overall survival overall survival from the time of primary cancer diagnosis (E) and from the time of leptomeningeal diagnosis (F). Multiple comparison (Figure I) shows significant differences in median OS between CNSide positive and cytology negative (1) versus CNSide and cytology negative (2) cohorts (*P* = .007), but no significant difference versus the CNSide and cytology positive (3) cohort.

**Figure 3.**
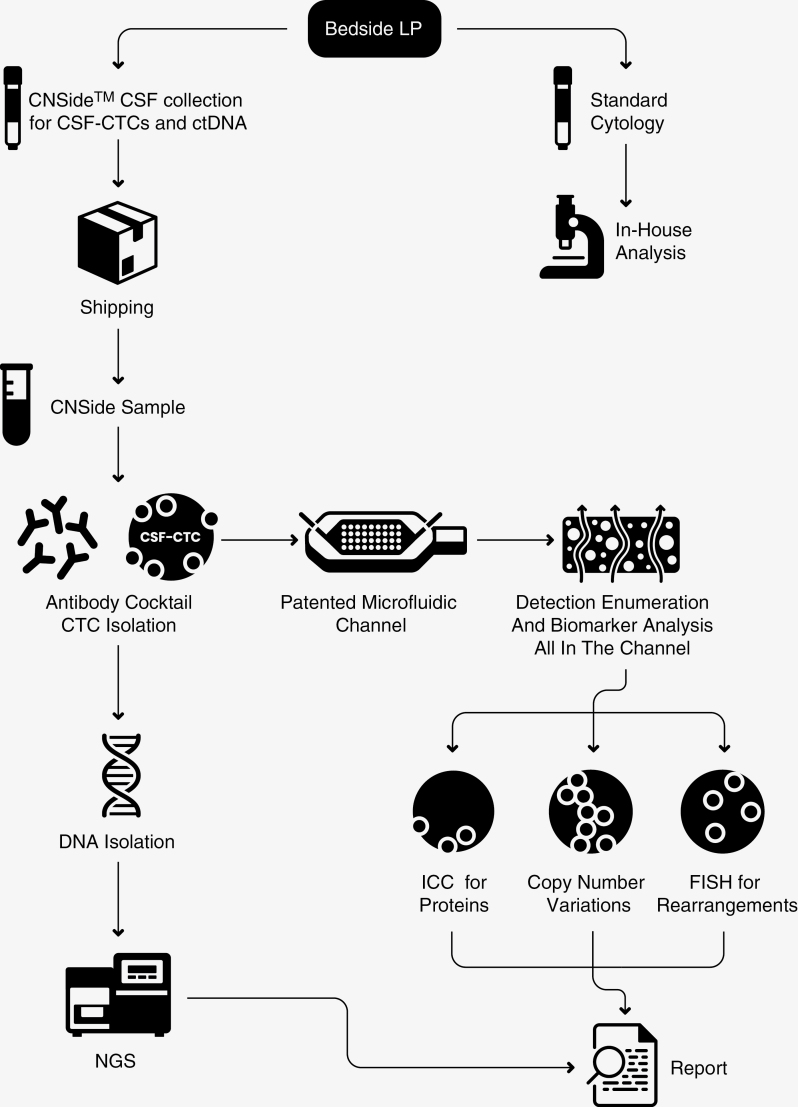
CSF sample workflow. A diagnostic lumbar puncture (LP) was performed at the bedside or occasionally via imaging guidance in the outpatient or occasionally inpatient settings. CSF was sent to cytology (volume 1–3 ml) and simultaneously shipped for CNSide testing (volume provided 2–10 ml, requested 5–8 ml). Upon receipt at the processing center, the CSF sample is divided for CTC isolation using a cancer-specific antibody cocktail. The CTC count and density preliminary report generated by the enumeration assay, followed by biomarker analysis including ICC for protein expression, FISH for rearrangements, and copy number variations. Simultaneously DNA isolation is tested for mutations by NGS methods.

Figure 2 shows the Kaplan–Meier overall survival curves for various subgroups and comparisons. The EANO-positive (confirmed, probable, possible) cohort had a median overall survival of 7.0 (95% CI: 4.0–13.0) months (Figure 2A). Median overall survival was 34.0 (95% CI: 14.0–49.0) and 17.0 (2.0–67.0) weeks for those with controlled versus uncontrolled systemic disease (*P* = .161) (Figure 2B). Median overall survival of cytology positive was 6.0 (95% CI: 2.0–11.0) weeks and 13.0 (95% CI: 5.0–20.0) weeks for cytology negative (*P* = 0.123, Figure 2C). Median overall survival from LMD diagnosis was 4.0 (95% CI: 2.0–7.0) weeks for CNSide™ positive and 16.0 (95% CI: 6.0–22.0) weeks for CNSide™ negative cases (Figure 2D). Median overall survival from LMD diagnosis did not significantly differ between primary breast or lung cancers, 10.0 (95% CI: 4.0–14.0) vs. 7.5 (95% CI: 2.0–19.0) weeks, respectively, *P* = 0.738 (Figure 2F), though the time between primary and leptomeningeal metastasis diagnosis was significantly longer for the breast compared to lung subcohort (Figure 2E). Median overall survival for patients treated with intrathecal chemotherapy (ITc) was 15.0 (95% CI: 1.0–NA) weeks compared to 3.0 (95% CI: 1.0–6.0) if deemed ineligible (*P* = .017, Figure 2G). OS median (95% CI) for patients on systemic therapy with low CNS penetrance at the time of LMD diagnosis was 27.0 (4.0–49.0) weeks, but surprisingly lower for those previously on agents with high CNS penetrance (13.5 (2.0–NA) weeks, *P* = .771) (Figure 3H). This may suggest that patients who develop LMD while already on CNS-penetrant regimens likely denote more refractory disease progression. Multiple comparison (Figure 3I) shows significant differences in median OS between CNSide positive and cytology negative (1/blue) versus CNSide and cytology negative (2/red) cohorts (*P* = .007), but no significant difference versus the CNSide and cytology positive (3/green) cohort (*P* = .997). There was a nonsignificant difference between the median OS between CNSide and cytology negative (2/red) and the CNSide and cytology positive (3/green) cohorts (*P* = .080).

There was no association between cell density by CNSide™ and mortality (HR:1.00, 95% CI: 0.99–1.00, *P* = .909). Survival was prolonged for cases eligible for intrathecal therapy (HR = 0.25, 95% CI: 0.08–0.79, *P* = .017), by definition, patients with improved prognostic factors like absence of hydrocephalus, CSF flow abnormality, extensive nodular disease, or otherwise extremely poor prognosis from their systemic disease.

## Discussion

The incidence and prevalence of LMD have increased over past decades due to increased detection bias (particularly improved diagnostic imaging) as well as advances in systemic therapies that have resulted in increased survival (but often lack maximal or sufficient CNS and especially CSF penetrance).^5,14,15^ Breast cancer is the most common histology in patients with LMD, with lobular subtype and triple-negative the most common risk factors.^5^ Prior surgical resection for brain metastases is associated with increased risk of leptomeningeal involvement, especially within the posterior fossa and resulting from a surgical spillover or drop metastasis of malignant cells into the leptomeninges induced by iatrogenic disruption of the blood-brain barrier.^5,15^ Early and accurate diagnosis of LMD, facilitated by advanced CSF analyses like CNSide^TM^, impacts diagnostic accuracy, facilitates proper prognostic estimates and discussions, and guides proper multimodal care.^15^ Wijetunga et al., in their proton craniospinal irradiation (pCSI) phase II trial cohort, showed that higher CSF ctDNA mean variant allele frequency (VAF) before pCSI (VAFpre) had worse OS (6 months for VAFpre ≥ 0.32 vs. 9 months for VAFpre < 0.32; *P* = .05). Similarly, increased VAF after pCSI portended worse survival (6 vs. 18 months; *P* = .008). Our cohort showed no similar correlation with pretreatment cell count density but warrants further validation in a larger prospective study. Serial CSF CTC and other assays also provide real-time and accurate response assessments that may in the future guide treatment changes and cessation by monitoring tumor dynamics and detection of molecular changes induced by treatment.

The increased utilization of blood-based liquid biopsies, specifically cfDNA, for molecular analysis in metastatic patients has served as an adjunct to tissue biopsies, and in some cases replaced repeat tissue biopsy altogether in patients who have declined surgical biopsy or deemed nonsurgical due to medical comorbidities. These liquid biopsies serve to monitor patients with known actionable molecular alterations not responding to targeted therapy and/or to further characterize concordance with primary genomic profiles. Tumor-specific genomic abnormalities can be detected in ctDNA using NGS techniques or PCR-based options, and have demonstrated a strong concordance with the genomic profile of malignant tissue, making ctDNA a potentially powerful biomarker.^3,11^ In a large-scale prospective concordance study of plasma and tissue NGS, Sugimoto et al. showed oncogenic alteration for non-small cell lung cancer was detected by plasma cfDNA sequencing in approximately 43% of patients and tissue assay in approximately 50% of patients. The concordance of plasma cfDNA sequencing compared with tissue DNA and RNA assays was 77% (EGFR, 78%; KRAS, 75%; BRAF, 85%; and HER2, 72%) and 47% (ALK, 46%; RET, 57%; ROS1, 18%; and MET, 66%), respectively.^16^ Of note, positive oncogenic drivers positive in plasma cfDNA and negative in tissue may result from unsuccessful genomic analysis from poor-quality tissue samples, while oncogenic drivers negative in plasma cfDNA and positive in tissue may result from the low sensitivity of cfDNA analysis or response to therapy.^16^ In patients with CNS disease, near-universal genomic divergence is seen across primary tumors and brain metastases with more than 50% of brain metastases harboring clinically actionable mutations not detected in the primary tumor.^17^

Concordance with plasma cfDNA and CSF cfDNA is less well-known, although some studies show distinct mutational diversity in ctDNA compared to plasma, but also increased genomic alterations.^18–22^ This discordance may be due to the purity of the CSF landscape, the intimate contact of tumor cells with the CSF and blood-brain barrier, as well as low systemic tumor burden.^18–20^ Discordance may partly result from patients with LMD receiving prior systemic therapy and/or radiation therapy causing selective pressures in the CSF cancer milieu.^18^ In our leptomeningeal cohort, CNSide™ detected a gain in KRAS (2, 5.6%) and EGFR (3, 8.3%) compared to the primary lung cancer profile, while a gain in ER (9, 23.1%), PR (3, 7.7%), and HER2 (2, 5.1%) and a loss of HER2 (3, 7.7.%) were noted compared to the primary breast cancer profile. Discordance was more common for primary breast cancer and overall showed gains in alterations rather than loss of expression. Potentially clinically informative alterations in the CSF can impact treatment decisions when selecting systemic therapy, however, most genomic alterations seen in the CNS cannot be targeted with currently available CNS-penetrant targeted therapies.^17,23^

CSF-based liquid biopsies offer a minimally invasive surrogate to the higher-risk neurosurgery required for brain and spine tissue biopsies and, as our study shows, can even increase the yield of standard CSF and MRI diagnostic tools with respect to leptomeningeal metastases. The increased sensitivity, retained specificity, quantitative circulating tumor cell analysis, and extensive genomic profiling provided by CNSide^TM^ typically required up to 8-10 mL of CSF, about 6–7% of the estimated 130–150 ml total volume of CSF in adults, and resulted in 1–3 weeks typically. Of note, the CNSide^TM^ assay provided a significantly higher detection rate compared to standard CSF cytology, increasing the diagnosis of LMD by 56.5%. The CNSide^TM^ assay provided extensive, tumor-specific, molecular data not available by standard CSF tests. Alternative paradigms exist for CSF qualitative and molecular profiling. *Genomic Testing Cooperative* (GTC) developed an approach to isolate cell-free total nucleic acid (cfDNA) followed by targeted NGS to sequence cell-free RNA (cfRNA) and cfDNA as novel liquid biopsy approach for both blood and CSF samples.^24^ GTC combines cfRNA and cfDNA NGS to evaluate mutations, fusion genes, and chromosomal structural abnormalities in liquid samples.^24^ cfRNA proved overall more sensitive than cfDNA in detecting mutations and, thus, reliable in detecting fusion genes and cfDNA is reliable in detecting chromosomal gains and losses.^24^ The liquid transcriptome, thus, can be used for diagnosis and staging, selecting therapy, predicting prognosis, and monitoring disease. Improved detection rates by these liquid biopsy strategies increase the certainty of LMD diagnosis, potentially enhancing prognostication and improving patient care by allowing for timely and appropriate radiotherapy, systemic therapy, and/or intrathecal chemotherapy selection. Optimizing multimodal and aggressive treatment for LMD diagnosis, especially earlier in the disease process before neurological disability or CSF flow abnormalities including hydrocephalus limit treatment options and prognosis, may improve survival for this rare but highly morbid leptomeningeal cancer diagnosis.^25–27^

## Conclusions

This retrospective, single-institution, and real-world analysis of CNSide™ showed increased sensitivity versus cytology and provided clinically relevant, cell-based molecular CSF analyses. The CNSide™ assay readily identified tumor cells circulating in the CSF, increasing the accuracy and potentially hastening LMD diagnosis, while also providing quantitative data on tumor burden and treatment response. The enumeration of CTC did not provide prognostic data in this cohort and future analyses with larger cohorts are necessary for prognostic estimates to guide treatment decisions. In the era of precision medicine, detection of actionable genomic alterations by using assays such as CNSide^TM^ and development of advanced trial designs and improved targeted intracranial penetrating therapies will help guide multimodal care for patients with otherwise dismal prognosis. Continued advancements in our understanding of LMD may provide a brighter outlook for these patients with poor prognosis, and it is imperative that neurocognitive decline and the patient’s welfare are at the forefront of any treatment decision.

## Supplementary Material

vdae071_suppl_Supplementary_Tables

## Data Availability

Research data are stored in an institutional repository and will be shared upon request with the corresponding author.
