# Supplementary material for: Comparative evaluation of the diagnostic and prognostic performance of CNSide™ versus standard cytology for leptomeningeal disease
Source: Neurooncol Adv. 2024 May 10;6(1):vdae071. doi: 10.1093/noajnl/vdae071 (PMC11217905; doi:10.1093/noajnl/vdae071)
Supplement: vdae071_suppl_Supplementary_Tables [file vdae071_suppl_Supplementary_Tables.docx]

**Supplemental Tables:**

| **Table S1 : Comparison of CNS Surgical vs. CSF Metastases Molecular Profiles** | | | | | | |
| --- | --- | --- | --- | --- | --- | --- |
| **ID** | **Primary** | **Surgery Date** | **Primary NGS Profile** | **CNS Tumor Profile** | **CNSide Positive** | **CNSide Profile** |
| 1 | Lung | 06/22/2018 | EGFR | NT | Y | Neg |
| 10 | Breast | 09/21/2020 | ER/PR | ER/HER 2 | N | IN |
| 19 | Breast | 06/22/2020 | ER/PR | ER/PR | N | IN |
| 25 | Lung | 08/02/2018 |  |  | N | Neg |
| 27 | Melanoma | 09/12/2018 | BRAF | BRAF | Y | Neg |
| 29 | Lung | 10/05/2020 |  | NT | N | Neg |
| 32 | Breast | 11/03/2016 | ER/PR | ER/PR | N | IN |
| 43 | Lung | 11/17/2021 | KRAS |  | Y | Neg |
| 51 | Breast | 04/26/2022 | ER/PR | ER/PR | N | IN |
| 52 | Astrocytoma | 07/14/2014 | MGMT; IDH | MGMT; IDH | N | Neg |
| 56 | Lung | 01/29/2019 |  |  | N | IN |
| 70 | Penile | 07/22/2022 |  | NT | N | Neg |
| 73 | Breast | 03/04/2021 | ER/Her2 | HER2 | N | IN |
| NA = Not Tested, Neg: Negative for Molecular, IN = Indeterminate | | | | | | |

| **Table S2: Breast Molecular Subgroup Analyses** | | | |
| --- | --- | --- | --- |
|  | **N** | **OS (95% CI)** | **Survival Curve** |
| **Primary Tumor** |  |  |  |
| HER2+ only | 25 | 10.0 (3.0, 17.0) | Curve 1 |
| ER/PR+ only | 7 | 7.0 (1.0, --) | Curve 2 |
| HER2 and ER/PR+ | 2 | 11.5 (6.0, --) | Curve 3 |
| Triple Negative | 3 | 7.0 (6.0, --) | Curve 4 |
| **CSF CNSide** |  |  |  |
| HER2+ only | 14 | 4.0 (1.0, 11.0) | Curve 5 |
| ER/PR+ only | 9 | 10.0 (3.0, --) | Curve 6 |
| HER2 and ER/PR+ | 7 | 7.0 (3.0, --) | Curve 7 |
| Triple Negative | --- | --- | N/A |
|  | | | |
| 1.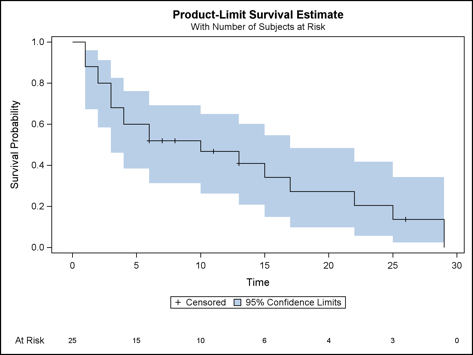 | | 2.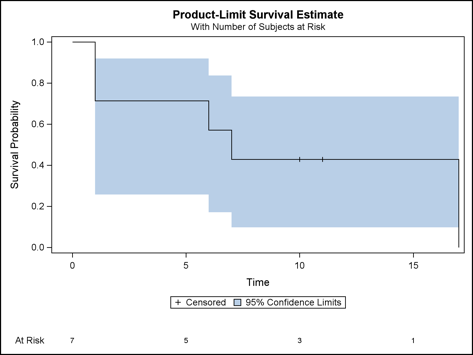 | |
| 3.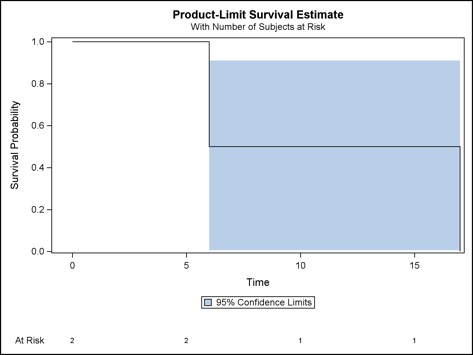 | | 4.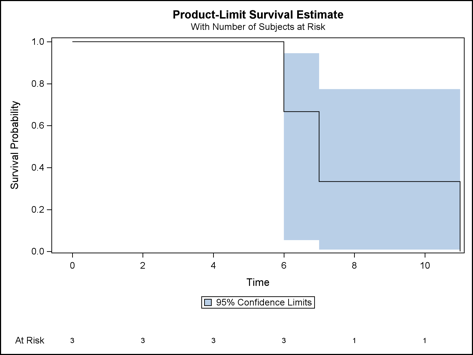 | |
| 5.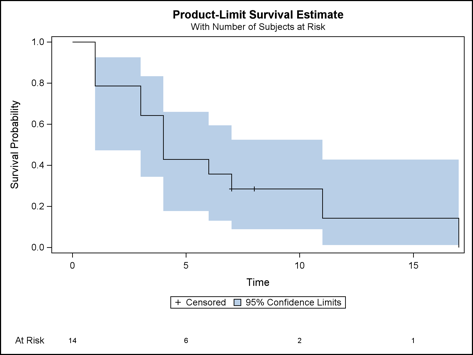 | | 6.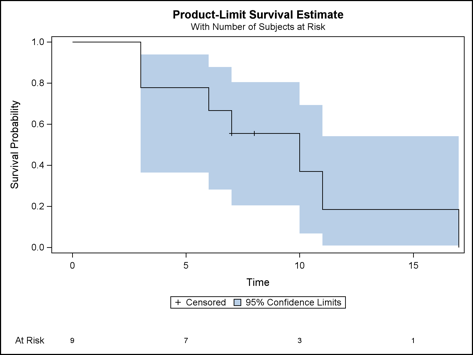 | |
| 7.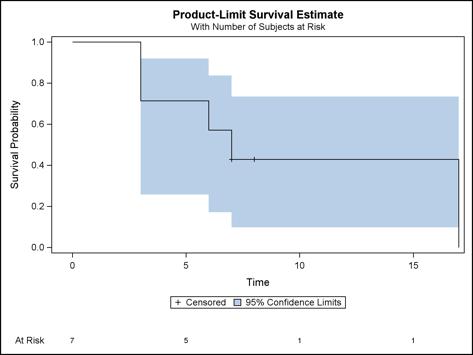 | | Survival analyses performed for each molecular subgroup: estrogen receptor (ER), progesterone receptor (PR), human epidermal growth factor receptor 2 (HER2) and/or positive (+) or negative by primary tissue (1-4) or CSF (5-7) analyses. | |
